# Language lateralization during the Chinese semantic task relates to the contralateral cerebra-cerebellar interactions at rest

**DOI:** 10.1038/s41598-017-14600-9

**Published:** 2017-10-25

**Authors:** Qing Gao, Zhongping Tao, Lintao Cheng, Jinsong Leng, Junping Wang, Chunshui Yu, Huafu Chen

**Affiliations:** 1 School of Mathematical Sciences, University of Electronic Science and Technology of China, Chengdu, 611731 China; 2 Center for Information in BioMedicine, Key Laboratory for Neuroinformation of Ministry of Education, Chengdu, 611731 China; 3 Information Technology Center, Chengdu Sport University, Chengdu, 610041 P. R. China; 4 Department of Radiology, Tianjin Medical University General Hospital, Tianjin, 300052 China; 5 School of Life Science and Technology, University of Electronic Science and Technology of China, Chengdu, 610054 China

## Abstract

Aiming to investigate whether handedness-related language lateralization is related to the intrinsic resting-state functional connectivity (RSFC) pattern within the language network, the present study integrated the information of functional activations during a semantic task of Chinese characters and FC in resting-state based on functional magnetic resonance imaging (fMRI) data of healthy left handers (LH) and right handers (RH). RSFC was calculated on a voxel-based level between the seed regions chosen from functional activations during the task and the rest of the brain. The results demonstrated that LH had significantly stronger RSFC than RH between the cerebellum and supratentorial areas of the frontal, parietal and temporal lobe, and between the occipital lobe and frontal/parietal lobe. Correlation analysis showed that RSFC values between right MFG and left cerebellum_crus2, between SMA and right cerebellum_crus2, and between the right cerebellum_crus1 and left MFG were negatively correlated with cerebral laterality index in LH and RH groups. Our results highlight key nodes of Chinese language brain network processing in the cerebellum, and suggest that atypical language dominance relates to stronger crossed reciprocal RSFC in the frontal-cerebellar system. The findings provide new insights into the intrinsic FC substrates underlying the atypical language lateralization of LH.

## Introduction

Language lateralization has been a subject of intense research^1^, and both behavioral and neuroimaging studies have shown that handedness contributes to language lateralization^2^. In recent years, the concepts of functional segregation and integration have been used to explore the underlying relationships of handedness and hemispheric dominance in the language brain network. Functional segregation allows localization of function. Researchers then revealed asymmetries of language-involved brain regions associated with handedness during certain language tasks, which were distributed in the frontal, temporal and parietal lobes with predominant left-lateralization in right-handers (RH) while a dominance reversal or a lower level of lateralization in left-handers (LH)^3–8^. Functional integration relates to communication among specialized brain regions. Further studies focused on the integration and interaction of distributed neural systems for certain language processing tasks. A number of studies demonstrated that handedness influences language network during language processing. For instance, one study described an altered modulation on the intra-hemispheric connections associated with handedness in semantic decision tasks^6^. In a word production task, LH showed connections significantly stronger than RH in right fusiform gyrus to bilateral Brodmann’s area 44^3^. Our previous study focused on the analysis of handedness and effective connectivity among brain regions recruited by a Chinese language task. This study found improved bihemispheric coordination and increased interhemispheric communication in LH^9^.

An increasing number of studies have focused on the intrinsic functional integration measured by spontaneous fluctuations in the brain at rest^10–12^. Task-evoked cerebral blood flow has been found to account for less than 5% of resting-state cerebral blood flow, which indicates that additional energy consumption associated with changes during task is remarkably small^12,13^. The intrinsic or task-free functional architecture of the brain involves abundant information for interpreting, responding to, and predicting environmental demands^13,14^. Semantic cognition depends on a distributed network^15^. Hence, integrating information of the brain’s functional segregation during explicit task and intrinsic functional integration may offer an improved understanding of the underlying neuromechanisms of semantic cognitive functions. Recent research on functional magnetic resonance imaging (fMRI) linked intrinsic functional connectivity networks with task-evoked activation in several cognitive domains^11,12,16–20^, such as working memory^16^, orthographic lexical retrieval^20^, Eriksen Flanker task^17^ and visual discrimination training^18^. The brain regions recruited by these cognitive functions are also engaged in active connectivity at rest^21^. Studies in language cognition proved the relation between semantic cognition in internal processes and intrinsic activity fluctuations; these studies indicated the feasibility of measuring language asymmetry using resting-state fMRI data^8,15,21–24^. Thus, resting-state functional connectivity (RSFC) networks provide novel insights into the organization of brain language networks. However, to our knowledge, only a few studies have integrated information of the brain’s intrinsic functional integration during resting-state and language-related task activations to reveal the association of RSFC and language lateralization in Chinese. Moreover, LH, who can be extremely informative in language lateralization studies, are paradoxically excluded in most analyses performed in this field; thereby the effect of handedness has been neglected in many studies.

The present study investigates whether the language hemispheric dominance of LH and RH is related to their RSFC language network pattern. We used the resting-state fMRI data and fMRI data of a semantic task of Chinese characters^9^. We hypothesized that the asymmetric language functional segregation associated with handedness could be reflected by the oscillatory integration in the language network at rest.

To define the nodes of the language network, the activated brain regions in healthy LH and RH groups in a Chinese semantic task were selected based on our previous study^9^. The differently activated areas between the two groups were chosen, which were considered associated with LH/RH specific language areas. The conjoined activated areas of the two groups based on conjunction analysis were also defined in our previous study^9^. These selected regions were included in the present study and acted as seed regions to calculate the FC between the seeds and the remaining brain voxels in resting-state. The differences of the FC maps of each seed between the LH and RH groups were compared. Correlation analysis was further performed to examine whether the altered RSFC between the two groups was associated with handedness-related language lateralization during the task performance.

## Results

### Chosen seed regions

**Figure 1:**
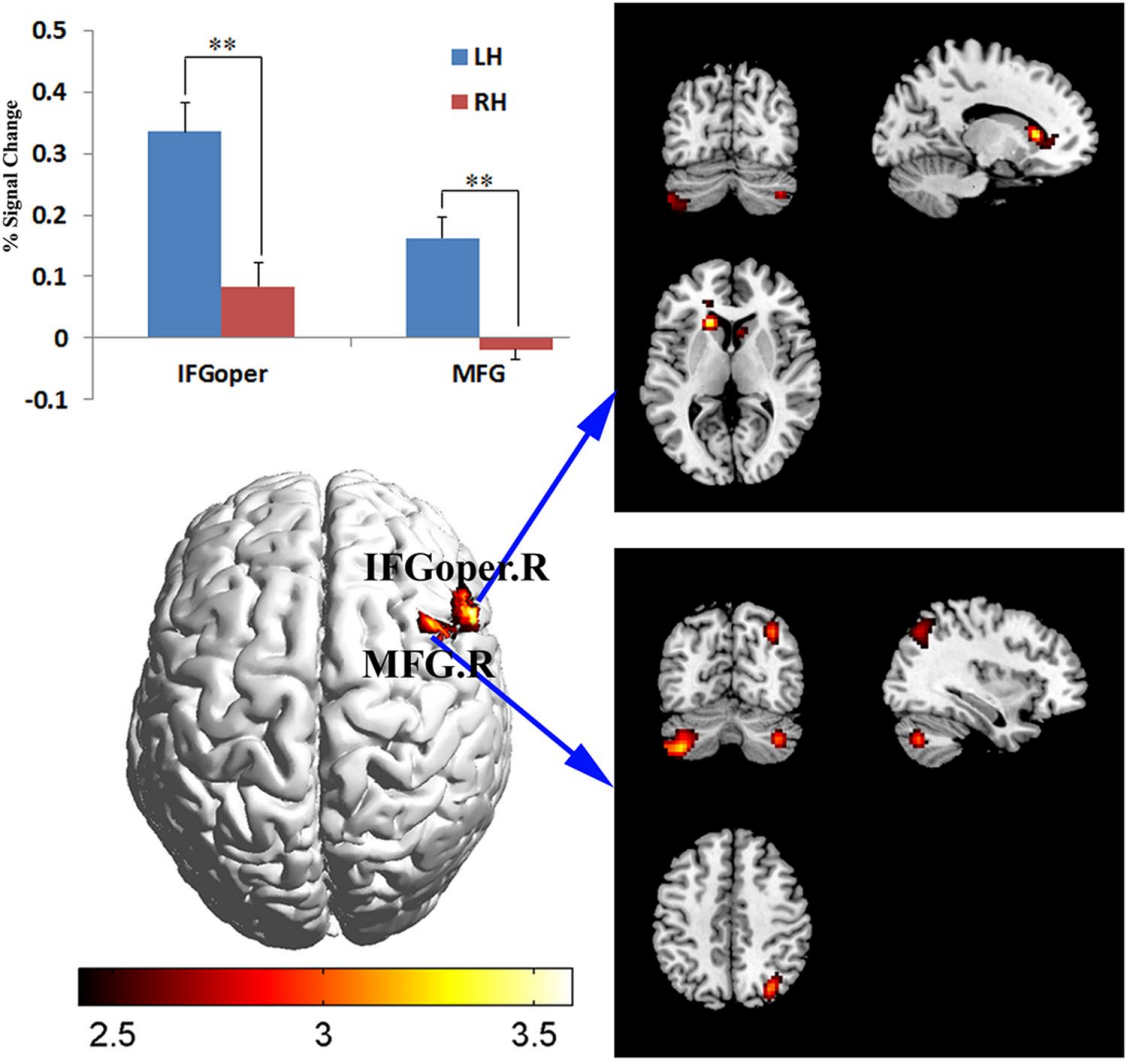
Left: significantly different activated areas between LH and RH groups (FDR *p* < 0.01) (**B**), and signal changes (%) in these areas (**A**). Right: significantly stronger RSFC in LH compared with RH (**C**: right IFGoper as seed; **D**: right MFG as seed). IFGoper, inferior frontal gyrus opercular; MFG, middle frontal gyrus; LH, left handers; RH, right handers; R, the right hemisphere.

The group analysis showed that two areas in the frontal lobe, namely the right middle frontal gyrus (MFG) and the inferior frontal gyrus opercular (IFGoper), displayed a significantly higher activity in LH than in RH. Figure 1 depicts the significantly different activated areas between LH and RH groups (Fig. 1B), along with the signal change (%) in these areas (Fig. 1A). The conjoint activated areas of the two groups during the task included the left MFG, left precentral gyrus (PreGC), supplementary motor area (SMA), bilateral insula (INS), left inferior occipital gyrus (IOG), bilateral middle occipital gyrus (MOG), and right cerebellum_crus1 and _crus2, as reported in our previous study^9^. Table 1 summarizes the details of the chosen seed regions, which were further used to calculate the RSFC between them and the remaining brain voxels.

**Table 1:** Chosen seed regions to calculate the RSFC between the seeds and the remaining brain voxels.

Region name	Abbreviation	Hem	Peak coordinates	Peak t-value
***Significantly differently activated areas***				
Middle frontal gyrus	MFG	R	(42 16 54)	3.49
Inferior frontal gyrus, opercular	IFGoper	R	(54 21 36)	3.72
***Conjoint activated areas***				
Middle frontal gyrus	MFG	L	(−51 18 36)	8.10
Supplementary motor area	SMA	L/R	(0 24 48)	8.62
Precentral gyrus	PreCG	L	(−51 9 45)	7.31
Insula	INS	L	(−33 24 −3)	7.19
		R	(30 24 −3)	4.99
Inferior occipital gyrus	IOG	L	(−36 −84 −12)	8.01
Middle occipital gyrus	MOG	L	(−18 −99 9)	6.20
		R	(24 −96 6)	5.43
Cerebellum_crus1	CRB_crus1	R	(18 −84 −21)	5.61
Cerebellum_crus2	CRB_crus2	R	(9 −84 −33)	6.19

### Significantly different RSFC between seeds regions and the remaining brain voxels

Figure 1 demonstrates the significantly stronger RSFC maps of seed right IFGoper (Fig. 1C) and seed right MFG (Fig. 1D) in LH than in RH (Alphasim corrected *p* < 0.01, cluster size = 67). Significantly stronger RSFC in RH than in LH was not detected. Table 2 summarizes the detailed results. For the case of seed right MFG, the brain regions with stronger RSFC in LH included right angular gyrus, left cerebellum_crus1, and bilateral cerebelum_crus2. By contrast, the brain regions of bilateral caudate nucleus and cerebellum_crus2 were reported for the case of seed right IFGoper (See Table 2 for details).

**Table 2:** Local maxima of significantly different RSFC between LH and RH in differently activated regions.

Region name	Abbreviation	Hem	Peak coordinates	Peak t-value
***Right middle frontal gyrus***				
Angular gyrus	ANG	R	(36 −72 45)	4.07
Cerebellum_crus1	CRB_crus1	L	(−39 −72 −36)	4.08
Cerebellum_crus2	CRB_crus2	L	(−39 −72 −45)	4.49
		R	(36 −69 −39)	3.94
***Right inferior frontal gyrus, opercular***				
Caudate nucleus	CAU	L	(−18 24 6)	4.86
		R	(6 15 3)	3.32
Cerebellum_crus2	CRB_crus2	L	(−48 −69 −45)	3.62
		R	(39 −72 −39)	3.74

**Figure 2:**
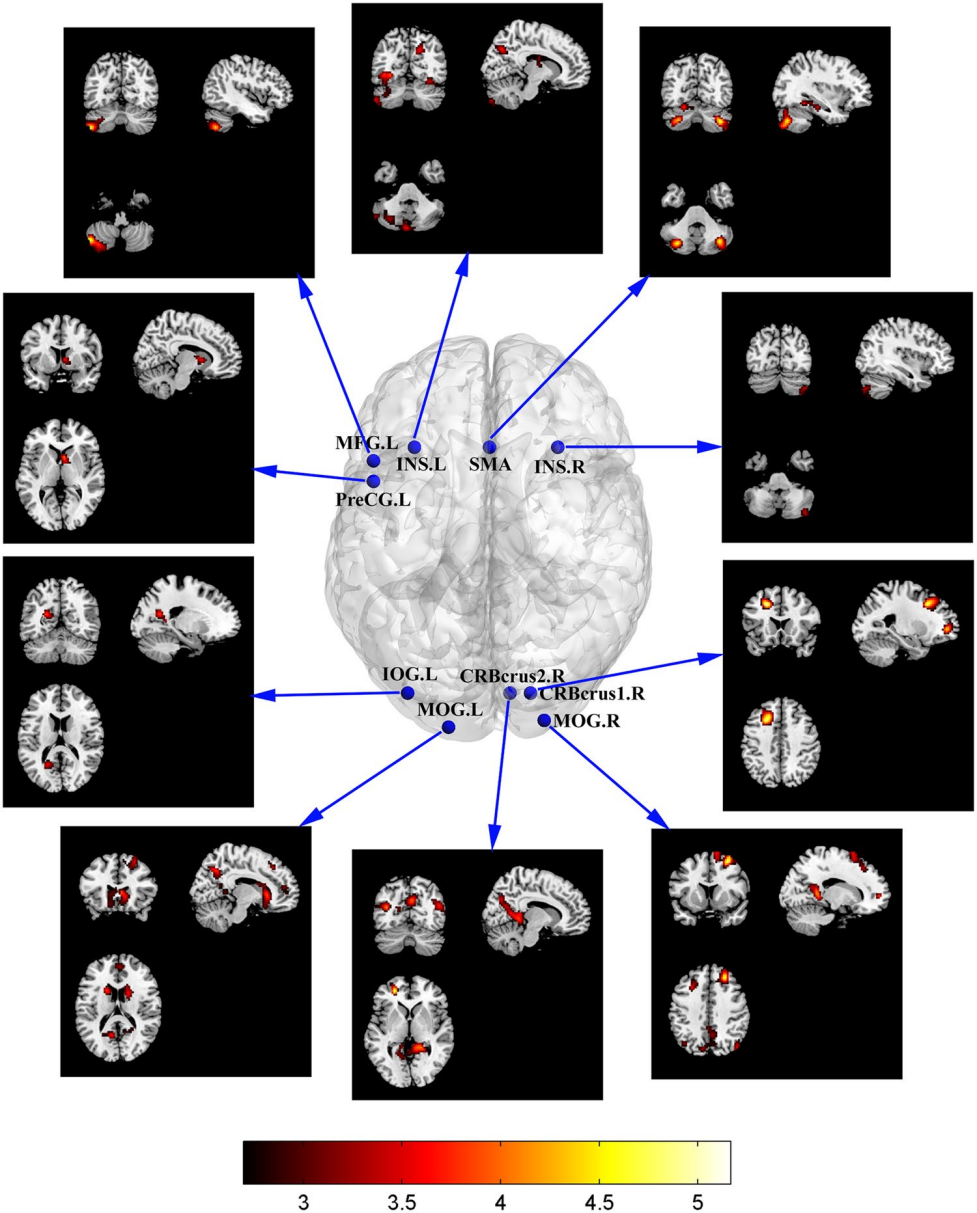
Significantly stronger RSFC in LH than in RH (conjoint activated areas as seeds, Alphasim corrected *p* < 0.01, cluster size = 67). CRBcrus1, cerebellum_crus1; CRBcrus2, cerebellum_crus2; INS, insula; IOG, inferior occipital gyrus; L, the left hemisphere; MFG, middle frontal gyrus; MOG, middle occipital gyrus; PreCG, precentral gyrus; R, the right hemisphere; SMA, supplementary motor area.

Figure 2 depicts the significantly stronger RSFC maps of seeds in conjoint activated areas in LH compared with those in RH (Alphasim corrected *p* < 0.01, cluster size = 67). Table 3 shows the details of the local maxima of significantly different RSFC between LH and RH groups. Most of the significantly stronger connections were long-range connections between the cerebellum and the supratentorial areas of the frontal, parietal and temporal lobe, and between the occipital and the frontal/parietal lobes. Significantly stronger RSFC in RH than in LH was not detected.

**Table 3:** Local maxima of significantly different RSFC between LH and RH in conjoint activated regions.

Region name	Abbreviation	Hem	Peak coordinates	Peak t-value
***Left middle frontal gyrus***				
Cerebellum_crus2	CRB_crus2	L	(−48 −63 −51)	4.78
***Supplementary motor area***				
Fusiform gyrus	FG	L	(−36 −48 −12)	3.99
Cerebellum_crus2	CRB_crus2	L	(−30 −72 −39)	4.53
		R	(36 −69 −42)	4.68
Cerebellum_6	CRB_6	L	(−21 −72 −18)	3.78
***Left precentral gyrus***				
Caudate nucleus	CAU	R	(6 12 3)	3.95
***Left insula***				
Cuneus	CUN	L	(−3 −78 36)	3.44
		R	(15 −66 36)	3.85
Fusiform gyrus	FG	R	(33 −48 −9)	4.44
Thalamus	THA	R	(9 −9 12)	3.98
Cerebellum_crus2	CRB_crus2	L	(−24 −78 −48)	4.55
***Right insula***				
Cerebellum_crus2	CRB_crus2	R	(42 −78 −42)	3.35
***Left inferior occipital gyrus***				
Precuneus	PCUN	L	(−15 −57 15)	3.77
***Left middle occipital gyrus***				
Middle frontal gyrus, orbital	MFGorb	R	(3 57 −12)	4.21
Superior frontal gyrus	SFG	R	(15 30 45)	3.64
Precuneus	PCUN	L	(−12 −72 33)	3.94
		R	(12 −60 39)	3.71
Inferior Occipital gyrus	IOG	L	(−18 −93 −9)	3.61
Caudate nucleus	CAU	L	(−15 21 3)	3.01
		R	(6 18 3)	4.41
***Right middle occipital gyrus***				
Middle frontal gyrus	MFG	L	(−27 21 42)	4.05
		R	(27 36 45)	4.86
Superior frontal gyrus	SFG	R	(27 15 57)	5.19
Superior frontal gyrus, medial	SFGmed	R	(6 57 0)	3.84
Precuneus	PCUN	R	(6 −45 6)	4.89
Angular gyrus	ANG	L	(−39 −69 39)	3.21
		R	(45 −75 33)	4.63
Middle occipital gyrus	MOG	L	(−39 −78 27)	4.04
***Right cerebellum_crus1***				
Middle frontal gyrus	MFG	L	(−21 18 39)	5.02
		R	(27 30 39)	3.15
Superior frontal gyrus	SFG	L	(−24 48 0)	4.38
Middle temporal gyrus	MTG	L	(−39 −63 21)	3.25
***Right cerebellum_crus2***				
Middle frontal gyrus	MFG	L	(−21 18 45)	3.54
Superior frontal gyrus	SFG	L	(−24 48 0)	4.85
Middle temporal gyrus	MTG	R	(48 −66 21)	3.94
Cuneus	CUN	R	(3 −72 30)	4.10
Precuneus	PCUN	R	(18 −54 15)	4.15
Superior parietal gyrus	SPG	R	(21 −78 51)	3.45
Parahippocampal gyrus	PHIP	R	(21 −42 −6)	5.01

**Figure 3:**
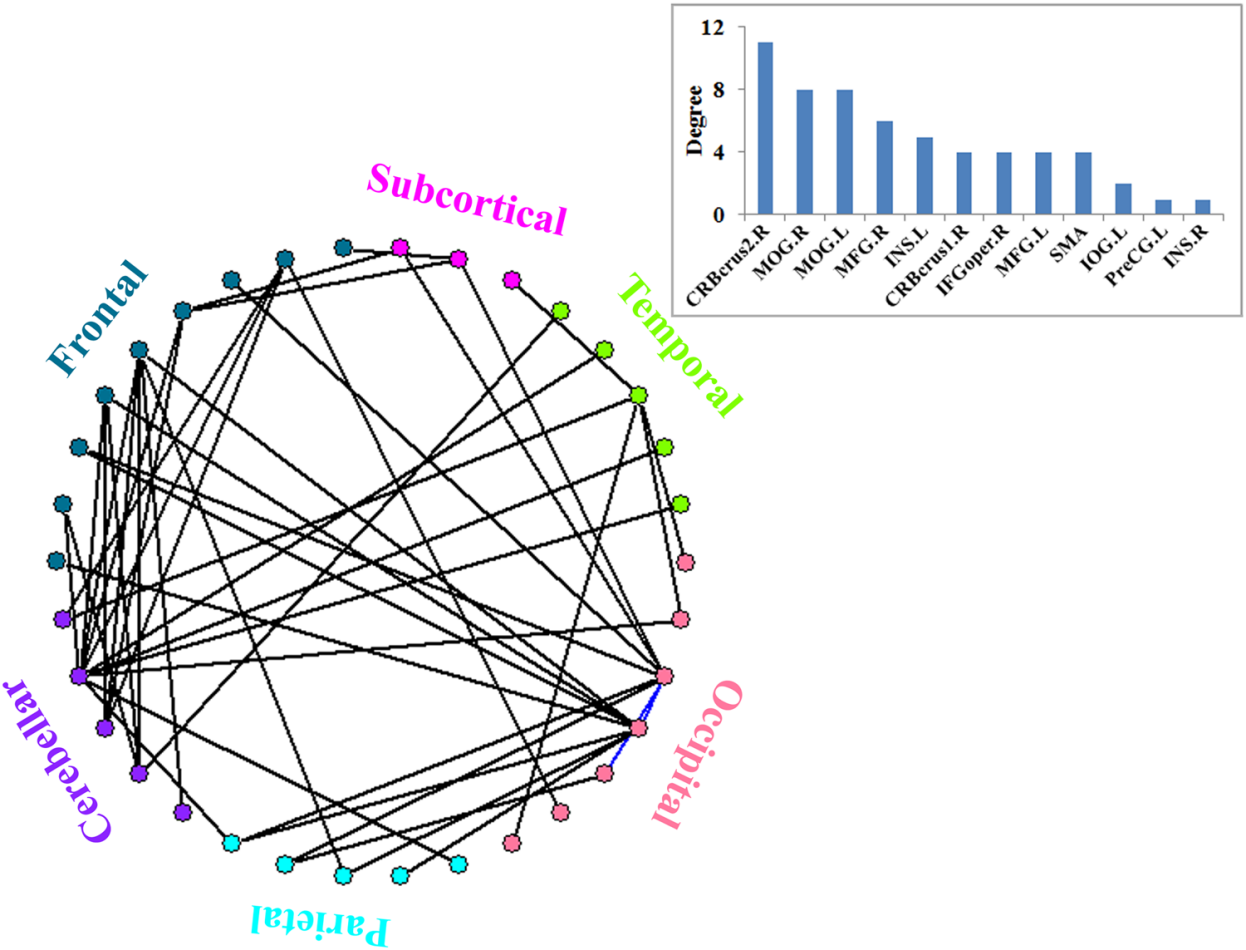
Schematic diagram of the RSFC difference between LH and RH groups. The two blue lines represent connections within the occipital lobe. All other lines indicate long-range connections, mainly distributed between the cerebellum and the supratentorial areas of the frontal, parietal and temporal lobes, and between the occipital and frontal/parietal lobes. Inset is the degree distribution of the seeds. The abbreviations are similar to those in Figs 1 and 2.

Figure 3 shows the diagrammatic representation of the significantly stronger RSFC of the chosen seeds in LH compared with those in RH. The two blue lines represent connections within the occipital lobe, whereas the black lines show long-range connections that are mainly distributed between the cerebellum and the supratentorial areas of the frontal, parietal and temporal lobes, and between the occipital and the frontal/parietal lobes. The degree of connectivity distribution of the seeds is shown as an inset. Right cerebellum_crus2 and bilateral MOG have relatively larger degrees of connectivity compared with the other seed regions.

**Figure 4:**
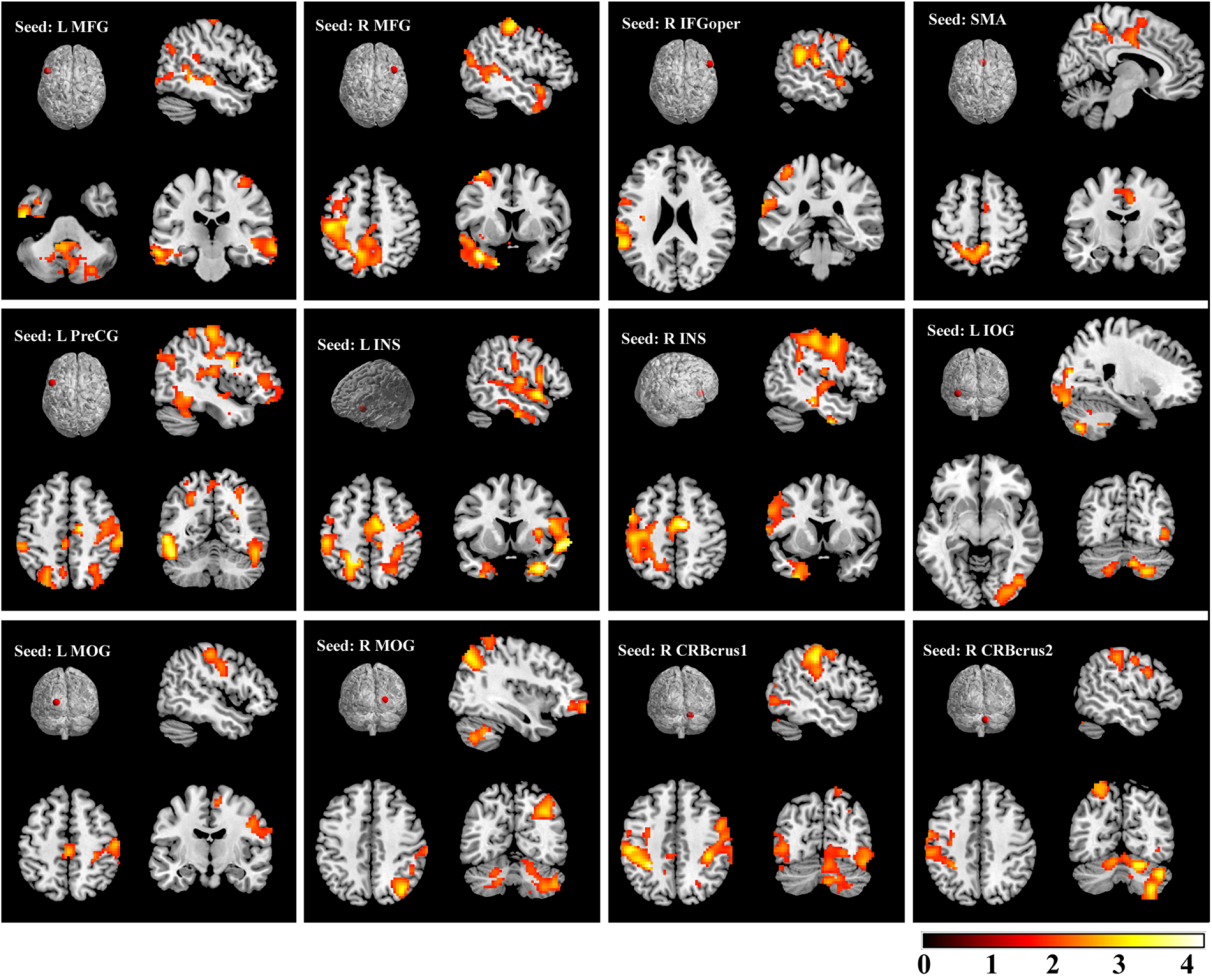
RSFC map of RH as a benchmark (Alphasim corrected *p* < 0.01). The abbreviations are similar to those in Figs 1 and 2.

We also present the RSFC map of RH as a benchmark in Fig. 4. The typical RSFC pattern demonstrated that the seed regions had connections with the Chinese semantic language areas of MFG, IFG, SMA, PreCG, postcentral gyrus, middle/superior temporal gyrus extending to temporal pole and INS, superior parietal gyrus, middle/superior occipital gyrus, precuneus, and the cerebellum.

### Correlations between RSFC and language lateralization

**Figure 5:**
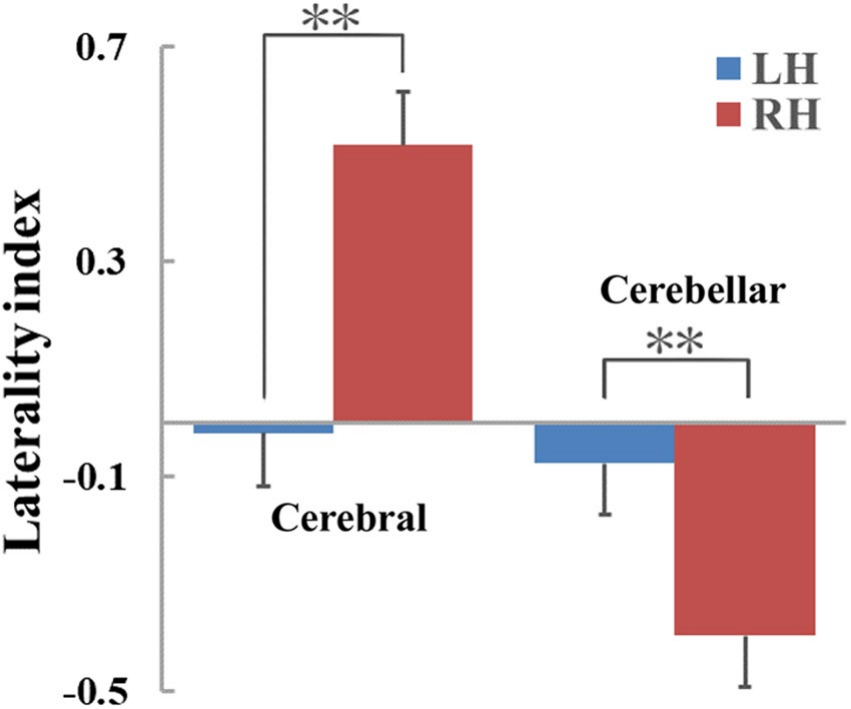
Group difference of the cerebral and cerebellar LI values between LH and RH. (**: significant threshold *p* < 0.0001).

Language lateralization was assessed by threshold-free laterality index (LI) and was tabulated based on the following categories: right-lateralized (LI ≤ −0.2), left-lateralized (LI ≥ 0.2), non-lateralized (−0.2 < LI < 0.2)^9,25^. Larger LI values represented increased left-lateralized activity patterns. All right-handed subjects had cerebral LI values larger than 0.2 in the cerebral cortex (0.52 ± 0.10, range 0.32~0.71). A total of 25 out of the 28 left-handed subjects showed cerebral LI values between −0.2 to 0.2; two had cerebral LI values larger than 0.2, and one had a cerebral LI value lower than −0.2. Figure 5 shows the group difference of the cerebral and cerebellar LI values between LH and RH. The mean cerebral LI for all left-handed subjects was −0.02 ± 0.11 (range −0.22~0.21). Significantly dissimilar cerebral LI was found between the two groups (*p* = 4e-26). In the cerebellum, 27 of the 28 RH had cerebellar LI values lower than −0.2, whereas one subject indicated non-lateralized pattern with a cerebellar LI value of −0.19. The mean cerebellar LI for all RH was −0.40 ± 0.10 (range −0.56~−0.19). In LH, 24 of the 28 LH had cerebellar LI values between −0.2 to 0.2, and four had cerebellar LI values lower than −0.2. The mean cerebellar LI for all LH was −0.07 ± 0.11 (range −0.34~0.11). Significantly disparate cerebellar LI was also found between the two groups (*p* = 3e-16).

**Figure 6:**
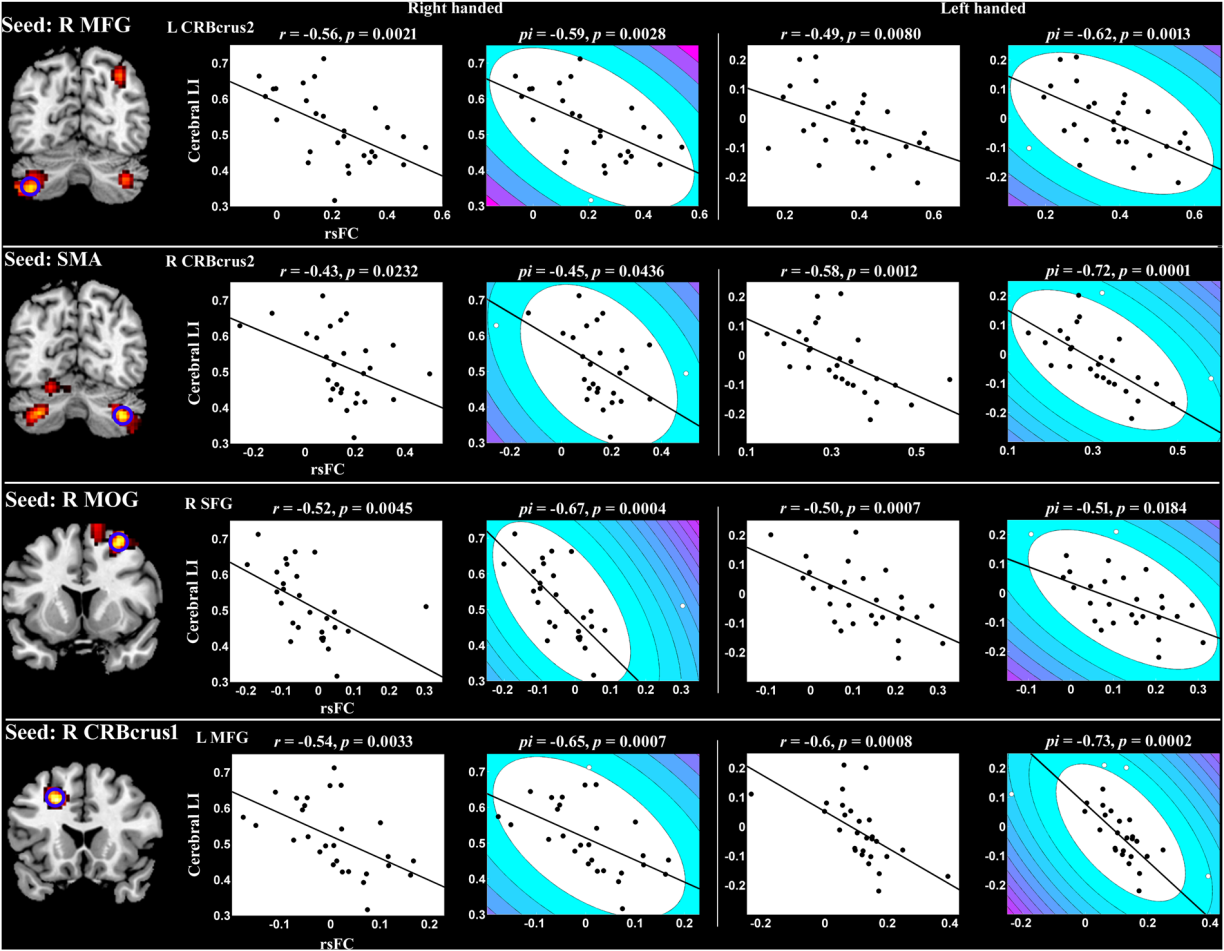
Significant correlations between RSFC and cerebral laterality index (LI) by Pearson correlation and Shepherd’s *pi* correlation analyses. The blue circle highlights the region whose significant correlation is depicted on the right. In the Shepherd’s *pi* correlation map, the contour lines indicate the bootstrapped Mahalanobis distance from the bivariate mean of the resampled data. The white points represent the outliers excluded from Shepherd’s *pi* correlation analysis. The abbreviations are similar to those in Figs 1 and 2.

Figure 6 depicts the significant correlations between RSFC and cerebral LI. However, the significant correlation between RSFC and cerebellar LI was not found. Correlation analyses were performed separately for LH and RH. The blue circle demonstrated the brain region showing significant correlations between its RSFC and cerebral LI. Pearson correlation analysis demonstrated that cerebral LI values were negatively correlated with RSFC association values between the right MFG and left cerebellum_crus2, between SMA and right cerebellum_crus2, between the right MOG and right superior frontal gyrus, and between the right cerebellum_crus1 and left MFG. Shepherd’s *pi* correlation was also applied to the data after outlier removal. The contour lines indicated the bootstrapped Mahalanobis distance from the bivariate mean of the resampled data. White points indicated outliers that were excluded from the correlation analysis.

## Discussion

The cerebral LI values of LH and RH individuals suggested that the brain activation pattern of RH was left lateralized, whereas the pattern of LH had a more bilateral distribution^9^. In the cerebellum, cerebellar LI values showed activated areas as right dominant in RH, which was contralateral to the activations in the cerebral cortex; whereas in LH, a more bilateral activity pattern was also found in the cerebellum compared with RH. The language lateralization patterns in LH and RH groups in our study were consistent with prior research, wherein LH presented a comparatively equally distributed language dominance between hemispheres in contrast to RH^9,26^. Moreover, our findings supported the idea of a bilateral recruitment of both the cerebral and cerebellar language areas in LH during Chinese semantic processing^9,27^, and suggested the concept of a lateralized linguistic cerebellum associated with handedness^9,28^. We also found that significantly different activations between LH and RH were located in right MFG and IFGoper. In fact, signal change analysis in these two areas demonstrated that significant activations in the LH group occurred in the right MFG and IFGoper, whereas no significant activation was detected in the RH group in these areas (Fig. 1A). The inferior and middle frontal gyri were activated and unique in Chinese semantic tasks comparing with English language tasks^29,30^ to complete the Chinese semantic access and judgment^30,31^. The bilateral involvement of the inferior and middle frontal gyri in LH and left-lateralized involvement of these areas in RH suggested that Chinese semantic processing in LH and RH had different patterns of representation in the frontal lobe^29,30,32,33^.

The typical RSFC pattern in RH indicated that the chosen seed regions had connections with the Chinese semantic language areas^30^. The dense language-related cerebello-cerebro-cortical functional connections in resting-state were detected. Significantly stronger RSFC pattern of the chosen seeds was found in LH compared with those in RH. Interestingly, most of the significantly stronger connections were in the type of long-range connections mainly distributed between the cerebellum and the supratentorial areas of the frontal, parietal and temporal lobes, and between the occipital and the frontal/parietal lobes. Right cerebellum_crus2 and bilateral MOG had relatively larger degrees of connectivity relative to other seed regions. This finding suggested that they may play an important role in understanding the relationship between language lateralization and RSFC in the language network.

Neuroanatomical studies proved that the prefrontal, parietal and temporal cortices demonstrate topographically organized projections to the cerebellum^34–36^. The anatomically cerebello-cerebro-cortical pathways have been suggested to provide a neural substrate for the cerebellum to actively and directly participate in cognitive and linguistic functions^9,34,36–38^. Our results further showed that the language-associated cerebello-cerebro-cortical functional connections in resting-state were influenced by handedness: bilateral cerebellum had significant stronger RSFC to the supratentorial areas including MFG, superior frontal gyrus, inferior frontal gyrus, SMA and INS in LH than in RH. This outcome suggested that handedness affected the cerebro-cerebellar system, such that left-handedness enhanced the connections in the system. More crossed reciprocal cerebello-cerebro-cortical connections between the language-associated regions may partly explain the consistently more symmetrical activated pattern in both the cerebral cortex and the cerebellum during the performance of Chinese semantic task in LH than in RH.

The correlation analysis in our study revealed that RSFC associations between specific brain regions were strongly related to the individual cerebral LI values in LH and RH groups. The results suggested that handedness-involved language lateralization related to crossed reciprocal connections in the frontal-cerebellar system in resting-state. Thus, the more atypical (trend to right hemisphere) the language lateralization was, the stronger the crossed reciprocal connections in the frontal-cerebellar system were. This result emphasized the importance of MFG, cerebellum_crus1 and cerebellum_crus2 in revealing the relation between language lateralization and RSFC^9,39^.

Occipital areas have been found to be consistently active during Chinese language tasks and have been suggested as specific nodes in the Chinese language network^30,31,33,40^. The areas were supposed to be relevant to the processing of the visual properties of Chinese characters and words^40^. Unlike English words, Chinese characters have a spatial structure, consisting of strokes or radicals that fit into a square-shaped space^30,41^. Bilateral MOG was revealed to be activated across three language-processing components including the orthographic, phonological, and semantic processing of Chinese characters^30^. This finding suggested that visuospatial analysis for the Chinese grapheme processing was needed for the three language-processing components including the semantic access^9,30,31,41^. In our study, LH had stronger RSFC between MOG and precuneus, and between MOG and right middle/superior frontal gyrus. Moreover, RSFC between the right MOG and superior frontal gyrus was found to be negatively correlated with cerebral LI values in both LH and RH. The results indicated stronger pathways for word form pattern extraction and later semantic access/judgment in LH, which were required in Chinese semantic processing^31,41^. This finding suggested that more atypical language dominance was associated with stronger functional connectivity between the right MOG and superior frontal gyrus. Handedness may influence the brain representation of language processing and the associated RSFC network. Along with findings that LH had more and stronger crossed reciprocal RSFC in frontal-cerebellar system compared with that of RH, the findings presumably provided RSFC clues for additional recruitment of right prefrontal areas for the Chinese semantic task in LH. Our findings may also lead to new interpretations of the fact that the severity of aphasia is milder in LH regardless of the hemisphere injured, and that recovery is more rapid and more complete in LH than in RH after an aphasia event^42^.

As demonstrated in our previous study, the RH group had the maximum Edinburgh Handedness Inventory (EHI) score of + 100, and the LH group had the minimum EHI score of −80 instead of −100. Social pressure for right-handed writing and eating is very strong in the Chinese population; thus, the born left-handed people are always forced to write and eat using their right hands^43,44^. The left-handed subjects recruited in our study reported to use their right hands to write and eat. This findings may bias the results in our study, but our results still have universal meaning in Chinese population because it is common in China^9,43,44^.

In summary, the present study integrated information regarding task activation and RSFC in the brain’s semantic processing network to investigate how language hemispheric dominance in LH and RH relates to the RSFC pattern. We found that LH had a larger RSFC language network than RH. Moreover, our task and resting-state studies unveiled novel cerebellar nodes in the Chinese language brain network. Our study expands the conclusion to suggest that the crossed reciprocal RSFC in frontal-cerebellar system may potentially be linked to the strength of hemispheric lateralization in LH and RH. Studies combining functional segregation during active semantic task and integration in resting-state will provide new insights into the organization of the brain language network associated with handedness.

## Methods

### Subjects

Participants consisted of 28 healthy left-handed subjects (17 females, age = 24.2 ± 2.5 years) and 28 healthy right-handed subjects (15 females, age = 24.7 ± 1.9 years). This study sample was previously analyzed in a task-related fMRI research of a study of our group^9^. The criterion for left handedness was an EHI score^45^ lower than −50, whereas the criterion for right handedness was an EHI score higher than 50^9^. The mean EHI score were −66.4 ± 13.4 for the left handedness group, and 96.8 ± 7.2 for the right-handedness group. The subjects were had no history of psychiatric or neurological illness, or prolonged impairment of function or language capability, and they had normal or corrected to normal vision. All subjects were native Chinese speakers and reported no exposure to the Korean language to ensure that Korean characters would serve as an appropriate perceptual control task. The protocol observed in the present study was approved by the local Ethics Committee of Tianjin Medical University and was conducted in accordance with the approved guidelines. Informed written consents were obtained from all participants.

### Experimental Paradigm

The experiment was performed on a 3.0-T GE Signa HDx MR scanner (Tianjin Medical University, Tianjin, China) using a gradient-recalled echo planar imaging (EPI) sequence with an 8-channel head coil. For the resting-state scans, subjects were instructed to simply rest with their eyes closed, not to think of anything in particular, and not to fall asleep. The acquisition parameters for functional imaging were as follows: TR = 3000 ms, TE = 30 ms, FOV = 22 cm, matrix = 64 × 64, voxel size = 3.44 × 3.44 × 4 mm^3^, 38 transverse slices with slice thickness = 3 mm, slice gap = 1 mm, and flip angle = 90°. The scan time for the resting-state fMRI and the language task were 6 min and 4 min, respectively. High-resolution 3D T1-weighted anatomical images were also acquired in sagittal orientation using a fast spoiled gradient recalled sequence (BRAVO, TR = 7.8 ms, TE = 3.0 ms, flip angle = 7°, matrix size = 256 × 256 × 176, slice thickness = 1mm without slice gap, and voxel size = 1 × 1 × 1 mm^3^).

In our previous study, we investigated the functional activity patterns and the effective connectivity network during a Chinese semantic task^9^. The fMRI experiment was a blocked design, with four semantic task blocks and four control task blocks alternatively. Each block lasted 30 seconds with 10 trials, and each trial included 200 ms for the “+” cue presented in the centre of the screen, 1800 ms for the meaning judgment task, and 1000 ms for the subjects to press the key. During the semantic task, participants were asked to judge if the two Chinese characters that appeared on the screen had the same meaning. During the control task, the participants were asked to judge whether two Korean characters were the same or not. Participants were requested to perform the tasks as quickly and accurately as possible. The Korean characters were randomly chosen, and half of the pairs were the same and the other half were different. The participants practiced a short version of the experimental task for familiarity. Subjects with accuracy rate higher than 80% during the practice were allowed to participate in the fMRI experiment.

### Data Analyses

Resting-state data and task data were preprocessed using statistical parametric mapping (SPM) software (SPM8, http://www.fil.ion.ucl.ac.uk/spm). The preprocessing of the images included slice-timing correction, head motion correction, and spatial normalization into a standard stereotaxic space with voxel size of 3 × 3 × 3 mm^3^ using the Montreal Neurological Institute (MNI) EPI template. A dataset with translational or rotational parameters exceeding ± 1 mm or ± 1° would be excluded. Recent studies demonstrated that functional connectivity analysis is sensitive to gross head motion effects^46,47^; thus, we further evaluated the framewise displacement (FD)^46^ in resting-state fMRI images to express instantaneous head motion, and a threshold of 0.5 was suggested. The largest FD of all subjects was less than 0.2 mm. Two sample t-test did not show a significant difference of FD between LH and RH groups (mean ± SD: 0.068 ± 0.038 for LH and 0.063 ± 0.037 for RH; *p* = 0.750).

The statistical parametric maps (*t*-statistics) of the contrast between the semantic condition and the control condition were generated using the general linear model. Two-sample *t*-test (FDR *p* < 0.01) was performed to obtain the differently activated regions of the LH and RH groups, with age, sex, education, head motion and EHI score as covariates. Signal changes of the differently activated regions were calculated using the MarsBaR toolbox (http://www.sourceforge.net/projects/marsbar).

The voxels with the highest statistical *t*-value in the regions were chosen as seeds to calculate the RSFC networks during resting-state. Seeds were also chosen from the conjoint activated areas of the LH and RH groups using conjunction analysis in SPM8, the results of which were reported in our previous study^9^. In the RSFC analysis, regions of interest (ROI) were defined as spheres with the centre at chosen seeds and the radius of 6 mm. The representative time series in each ROI was obtained by averaging the fMRI time series across all voxels in the ROI.

### Language lateralization assessment

The threshold-free LI on SPM *t*-maps was calculated for each subject to assess language lateralization^9^. Nagata *et al*. proposed that the approach could minimize the influence of the statistical threshold on LI assessment^48^. This approach calculates the number of left and right hemisphere voxels activated for the task relative to the baseline at a range of different statistical thresholds, then searches for the optimal regression function, which describes the relationship between the number of voxels and the statistical threshold^48,49^. The regression provides a constant term that is used to calculate a normalized difference between the left and right hemisphere activity^48,49^. A positive value of LI represents left-hemisphere dominance, whereas a negative value indicates right-hemisphere dominance^6^. We calculated LI for the cerebral cortex and the cerebellum, respectively. Hemispherical masks were employed to identify left or right hemispherical voxels. As suggested in previous studies, language lateralization was tabulated based on the following categories: right-lateralized (LI ≤ −0.2), left-lateralized (LI ≥ 0.2), and non-lateralized (−0.2 < LI < 0.2)^25^.

### Functional connectivity networks

Seed-based, voxel-wise RSFC was calculated by REST software (www.restfmri.net). Each time series was first corrected by linear regression to remove the possible spurious variances including six head motion parameters, white matter, and ventricular signals averaged from a white matter mask and a ventricular mask, respectively^50,51^. The residuals of these regressions were temporally band-pass filtered (0.01 < *f* < 0.08 Hz) to reduce low-frequency drifts and physiological high-frequency respiratory and cardiac noise^10^; they were also linearly detrended for further RSFC analysis. RSFC was calculated between the seed regions and the remaining brain on a voxel-based level for each subject using Pearson’s correlation. A Fisher’s r-to-z transformation was applied to the correlation matrices to improve the normality of the correlation coefficients. A spatial smoothing filter was subsequently employed for each volume by convolving with an isotropic Gaussian kernel (FWHM = 8 mm) before group comparison. Two-sample *t*-test was then utilized to obtain the regions with different functional connectivity to the seeds between the two groups, with age, sex and FD as covariates. The statistically significant threshold was set for multiple comparisons at the cluster level with Alphasim corrected *p* < 0.01 (cluster size = 67).

### Correlation analysis

To examine if the altered RSFC between the two groups were associated with handedness, relationships between RSFC values in regions showing significant group differences and cerebral/cerebellar LI values were further detected by Pearson correlation analysis, as well as Shepherd’s *pi* correlation analysis as a supplement^52^. Correlation analyses were separately performed for LH and RH. Shepherd’s *pi* correlation (http://www.fil.ion.ucl.ac.uk/∼sschwarz/Shepherd.zip) was further used because it is more robust to the presence of influential outliers and less strict than other correlation approaches in situations where a relationship is likely^52^. The bootstrapped Mahalanobis distance from the bivariate mean of the resampled data was calculated to exclude the influential outliers from the correlation analysis^52^. Shepherd’s *pi* correlation was then applied to the data after outlier removal^52^.
